# Impact of PCSK9 inhibitors in glycaemic control and new-onset diabetes

**DOI:** 10.1186/s12933-023-02077-y

**Published:** 2024-01-03

**Authors:** Ana M. González-Lleó, Rosa M. Sánchez-Hernández, Núria Plana, Daiana Ibarretxe, Pere Rehues, Josep Ribalta, Dídac Llop, Ana M. Wägner, Lluís Masana, Mauro Boronat

**Affiliations:** 1 Sección de Endocrinología y Nutrición. Complejo Hospitalario Universitario Insular Materno-Infantil de Gran Canaria (CHUIMI), Las Palmas de Gran Canaria, España; 2 Instituto Universitario de Investigaciones Biomédicas y Sanitarias (IUIBS), Universidad de Las Palmas de Gran Canaria (ULPGC), Las Palmas de Gran Canaria, España; 3 Universitat Rovira i Virgili. Unidad de Medicina Vascular y Metabolismo. Unitat de Recerca Lipids i Arteriosclerosi. Hospital Universitari Sant Joan, IISPV: CIBERDEM., Reus, España

**Keywords:** New-onset Diabetes Mellitus, Prediabetes, PCSK9 inhibitors, Real-life study, Hyperglycaemia, Familial hypercholesterolemia

## Abstract

**Background:**

The diabetogenic effect of statins has been well established by clinical trials, Mendelian randomisation studies and meta-analyses. According to large clinical trials, PCSK9 inhibitors (PCSK9i) have no deleterious impact on glucose metabolism. However, few real-life studies have yet evaluated the long-term effects of these drugs on glucose homeostasis and their impact on new-onset diabetes (NODM).

**Methods:**

We studied 218 patients treated with either alirocumab or evolocumab (70% with familial hypercholesterolemia) for at least three years (PCSK9iG). We studied the NODM rate in the nondiabetic group at baseline (168) and overall glucose metabolism control in the whole group. Incidental DM was compared with two groups. The first was a propensity score matching (PSM)-selected group (n = 168) from the database of patients attending the Reus lipid unit (Metbank, n = 745) who were not on PCSK9i (PSMG). The second was a subgroup with a similar age range (n = 563) of the Di@bet.es study (Spanish prospective study on diabetes development n = 5072) (D@G). The incidence was reported as the percentage of NODM cases per year.

**Results:**

The fasting glucose (FG) level of the subjects with normoglycaemia at baseline increased from 91 (86-95.5) to 93 (87–101) mg/dL (*p* = 0.014). There were 14 NODM cases in the PCSK9i group (2.6%/y), all among people with prediabetes at baseline. The incidence of NODM in PSMG and D@G was 1.8%/y (*p* = 0.69 compared with the PCSK9iG). The incidence among the subjects with prediabetes was 5.1%/y in the PCSK9iG, 4.8%/y in the PSMG and 3.9%/y in the D@G (*p* = 0.922 and *p* = 0.682, respectively). In the multivariate analysis, only the FG level was associated with the development of NODM in the PCSK9iG (OR 1.1; 95% CI: 1.0-1.3; *p* = 0.027). Neither FG nor A1c levels changed significantly in patients with DM at baseline.

**Conclusion:**

A nonsignificant increase in NODM occurred in the PCSK9iG, particularly in patients with prediabetes, compared with the PSMG and D@G groups. Baseline FG levels were the main variable associated with the development of DM. In the subjects who had DM at baseline, glucose control did not change. The impact of PCSK9i on glucose metabolism should not be of concern when prescribing these therapies.

**Supplementary Information:**

The online version contains supplementary material available at 10.1186/s12933-023-02077-y.

## Background

The interest in proprotein convertase subtilisin/kexin type 9 (PCSK9) as a lipid-lowering target arose at the beginning of the present century after the identification of several families with familial hypercholesterolemia (FH) who carried gain-of-function mutations in the gene encoding PCSK9. The subsequent observation that loss-of-function gene variants were associated with reduced low-density lipoprotein (LDL) cholesterol (C) levels and fewer cardiovascular events led to its consideration as a potential drug target [1, 2].

Circulating LDL binds to the LDL receptor (LDLR) to form a complex that is internalised in the cell, where the LDL particles are degraded and the LDLR is recycled. PCSK9 is synthesised in the liver and released into plasma, where it also binds LDLR. When internalisation of the LDL-LDLR complex occurs in the presence of PCSK9, LDLR is also degraded, preventing recycling and reducing the expression of LDLR on the cell surface, mainly in hepatocytes.

In 2015, the Food and Drug Administration (FDA) and European Medicines Agency (EMA) approved the clinical use of alirocumab and evolocumab, two PCSK9 inhibitor monoclonal antibodies (PCSK9i). These drugs block the circulating PCSK9 protein, allowing the recycling of the receptors, thus increasing the availability of LDLR. These therapies lower LDL-C concentrations by approximately 60% and significantly reduce cardiovascular risk when added to statin therapy [3, 4].

Data from the Jupiter study [5], various subsequent meta-analyses [6] and results from Mendelian randomisation studies [7] have demonstrated the diabetogenic potential of statins, the cornerstone of lipid-lowering treatment, although the exact mechanism through which this phenomenon is produced is unknown. Various hypotheses have been formulated, such as their association with the decrease in LDL concentrations, the disturbed intracellular metabolism in pancreatic beta-cells induced by the hyperexpression of LDLR on the cell membrane and the subsequent increase in intracellular cholesterol [8, 9], or even body weight increase associated with statin treatment [10]. Given that PCSK9i notably raise LDLR expression and achieve much higher LDL-C reductions than statins, it would be reasonable to think that these new drugs might also have effects on glucose metabolism [11]. Large-scale clinical studies conducted to date have not observed a higher incidence of diabetes mellitus (DM) among participants treated with PCSK9i [12, 13]. However, Mendelian randomisation studies [7, 9], some meta-analyses [14, 15] and real-life studies [16] published in recent years have found a slight deterioration in glycaemic control among users of these drugs.

The aim of this study was to examine the development of glucose metabolism disorders and new-onset DM in patients with hypercholesterolemia receiving treatment with PCSK9i.

## Methods

### Study design and population

This is a retrospective observational study based on real clinical practice. Two hundred eighteen patients over 18 years of age on PCSK9i, because of clinical indication, for a mean follow-up of 3.2 years, from two lipid units at University Hospitals in Las Palmas de Gran Canaria and Reus (Spain), were included in the study (PCSK9iG). Subjects who discontinued treatment before the first year and those who were lost to follow-up were excluded. To compare the incidence of new-onset diabetes, the nondiabetic patients at baseline (n = 168) were compared to a similar group of patients not taking PCSK9i selected by a propensity score matching technique (PSM) from the database (Metbank, n = 745) of patients enrolled in the Reus Lipid Unit because of metabolic disturbance. The mean follow-up of this group was 6.6 years. The incidence of new-onset DM was also compared with that of the Di@bet.es study, a prospective, population-based study including 5072 participants, aimed at estimating the prevalence and incidence of DM in Spain (D@G) [17]. For comparison with the PCSK9iG, a subgroup of 563 subjects with a similar age were selected with a mean follow-up of 7.5 years.

Medical records of PCSK9iG participants were reviewed, and demographic data, personal history of DM and cardiovascular disease (CVD) (defined as acute myocardial infarction, angina pectoris, coronary revascularization, ischaemic stroke or peripheral vascular disease), type of hypercholesterolemia (FH, polygenic hypercholesterolemia or mixed dyslipidaemia), and time and characteristics of lipid-lowering treatment were compiled. Anthropometric data (height and weight) were also recorded at baseline and at the end of follow-up. Initial and final body mass index (BMI) and weight change during the time of exposure to PCSK9i were obtained. Standard biochemical data, including LDL-C, lipoprotein (a) and glycaemic profile [fasting glucose (FG) and glycated haemoglobin (A1c)] were recorded at baseline and follow-up. The new-onset DM rate was determined according to the American Diabetes Association (ADA) guidelines and expressed as a percentage per year (%/y) in the PCSK9iG, PSMG and D@G groups.

PCSK9iG patients received alirocumab 75 or 150 mg or evolocumab 140 mg every two weeks at the discretion of their physicians. Depending on the status of their glucose metabolism prior to PCSK9i treatment, participants were classified into three categories according to ADA definitions: normoglycaemia (FG < 100 mg/dl and A1c < 5.7%), prediabetes (pre-DM) (FG between 100 and 125 mg/dL and/or A1c between 5.7 and 6.4%) and DM (FG ≥ 126 mg/dL and/or A1c ≥ 6.5% on two or more occasions, or use of hypoglycaemic medication). The same criteria, observed in at least one blood test, were used to determine category changes of the patients during treatment.

### Statistical analysis

Descriptive data are presented as the mean +/- standard deviation or median (interquartile range) for quantitative variables and as percentages for categorical variables. The groups were compared using ANOVA/Student’s ‘t’ test or the Kruskal‒Wallis/Mann‒Whitney test for quantitative variables, depending on whether the distribution was normal or not and according to the number of groups analysed. To compare follow-up with baseline results, Student’s ‘t’ test for related data or Wilcoxon’s test was used, depending on whether the distribution was normal or not.

A propensity score matching (PSM) is a statistical technique that was designed to control for potential clinically relevant confounding variables and effectively balance the distribution of covariates between the groups to minimize bias and enhance the validity of our comparative analysis. The propensity score is the probability of receiving the treatment given a set of observed covariates of each individual selected, which is obtained using logistic regression analysis. The idea is to create a pseudo-randomised group not on PCSK9 inhibitors that is comparable to the treatment group based on the observed covariates. Thus, PSM involves pairing individuals from the treatment group with similar propensity scores to individuals from the control group. The matching process was carried out with the MatchIt R package. Patients were selected from the database (Metbank, n > 745) of patients attending the lipid units because of dyslipidaemia and/or associated disturbances, such as DM, obesity or metabolic syndrome. Subjects without baseline DM from the Metbank and PCSK9iG cohorts were matched at a 1:1 ratio. The covariates used as predictors in this matching process included age, sex, BMI, FG levels, statin use and FH diagnosis. A1c was not included in the matching process because measurements were unavailable for a considerable number of patients, and its inclusion would compromise the optimality of the process. These covariates were selected because they were identified as potential confounders, and, moreover, they were measured in all patients.To compare the proportion of patients who developed DM during follow-up, in the PCSK9iG, PSMG and D@G groups, a two-proportion z test, which assesses whether there is a significant difference between two known proportions, was used. It is a test commonly employed when dealing with categorical data, and the goal is to assess whether the proportions in the two groups are significantly different from each other.

Finally, to identify the factors associated with the development of DM, a multivariate logistic regression analysis was performed in the non-DM PCSK9iG. FG, A1c, age, sex, baseline BMI, FH diagnosis, PCSK9i type, exposure to treatment time, centre of origin, concomitant treatment with statins, and percentage reduction in LDL-C were included as independent variables. Odds ratios (OR) along with their corresponding confidence intervals (CI) were calculated to assess the impact of the mentioned variables on the onset of new DM. SPSS version 21.0 for Windows (IBM Corporation, Armonk, NY, USA) and RStudio (version 4.0.1) were used to perform the analyses. A *p* value less than 0.05 was considered significant.

## Results

Two hundred eighteen patients were included in the PCSK9iG group, and 53.2% of these patients were men. The patients were overweight, and the median age was 62 years (54–69). Table 1 shows the clinical characteristics of the PCSK9iG patients sorted by glycaemic status at baseline. A total of 70.6% of patients had FH, and more than half already had established CVD. Over two-thirds (68.8%) of the population used alirocumab (37.6% were on the 75 mg dose), and 31.2% used evolocumab. The rates were different between the two centres (Supplementary Table 1). The mean follow-up was 38.2 months [21.3–61.6]. After starting PCSK9i, a reduction in LDL-C of 57% [40.5–67.8] was achieved at six months of follow-up, and a reduction of 60% [43.5–70.7] was achieved after three years. One hundred and sixty-eight participants did not have DM (though 77 had pre-DM) at baseline. Participants with DM at the beginning (n = 50) were older and had a higher prevalence of ECV and BMI than non-DM subjects. Table 2 compares the main clinical characteristics of the non-DM PCSK9iG, PSMG and D@G groups.

**Table 1:** Characteristics of the PCSK9iG subjects according to their initial glucose metabolism status

	Total n = 218	Normoglycaemia n = 91	Pre-DM n = 77	DM n = 50	*p*
Age (years)	62 (54–69)	59 (51–67)	62 (54–69)	66 (58–72)	**0.001**
Sex (male, %)	53.2	58.2	45.5	56	0.230
Baseline BMI (kg/m^2^)	29 ± 4.6	27.7 ± 4.5	29.8 ± 4.8	30.4 ± 3.9	0.001
Final BMI (kg/m^2^)	29 ± 4.7	27.8 ± 4.7	29.7 ± 4.8	30.1 ± 4.3	0.004
CVD (%)	53.2	45.1	48.1	76	**0.001**
FH (%)	70.6	69.2	76.6	64	0.289
Ezetimibe (%)	62.8	56	68.8	66	0.202
Statins (%)	73.4	70.3	77.9	72	0.523
PCSK9i starting dose (%)					**0.005**
Al 75 mg Al 150 mg E 140 mg	37.6 31.2 31.2	34.1 23.1 42.9	40.3 31.2 28.6	40 46 14	
Lp(a) (mg/dL)	40.6 (11.2–98.8)	57.4 (10.6-100.6)	23 (9.2–84)	48 (14–108)	0.509
LDL-C (mg/dL)	158.1 (130.8-191.1)	157 (129–190)	173.3 (141.9-202.6)	153.1 (127.5-172.2)	**0.009**
Fasting glucose (mg/dL)	100 (91–114)	91 (86-95.5)	107 (100–112)	130 (114–160)	**< 0.001**
A1c (%) N = 151	5.9 (5.6–6.4)	5.5 (5.3–5.6)	5.8 (5.6–6.1)	6.7 (6.4–7.8)	**< 0.001**

BMI: body mass index; CVD: cardiovascular disease; FH: familial hypercholesterolemia; Lp(a): lipoprotein A; LDL-C: LDL cholesterol; A1c: glycated haemoglobin; preDM: prediabetes; DM2: type 2 diabetes mellitus

**Table 2:** Matching baseline characteristics of the non-DM subjects from the three compared groups

	PCSK9iG (n = 168)	PSMG (n = 168)	Di@bet.es study (n = 563)
Age (years)	59.5 ± 10.6	65.4 ± 11.8	64.5 ± 10.5
Sex (male, %)	52.4	50.8	39.7
BMI (kg/m2)	28.6 ± 4.7	28 ± 4.5	27.5 ± 4.7
Fasting glucose (mg/dL)	98.2 ± 12.4	95.6 ± 11.2	91.9 ± 12.6
A1c (%)	5.7 ± 0.4	6.2 ± 6.3 (n = 96)	N/A
FH (%)	72.6	72.6	N/A

BMI: body mass index, FH: familial hypercholesterolemia; A1c: glycated haemoglobin; DM2: type 2 diabetes mellitus

After a mean follow-up of 3.2 years, the non-DM patients at baseline in the PCSK9iG group experienced a slight nonsignificant increase in FG (97 (90–107) vs. 99 (90.3–107) mg/dL, *p* = 0.058). Twenty-six of the 91 subjects with normoglycaemia (28.6%) progressed to pre-DM, but none developed DM.

**Fig. 1:**
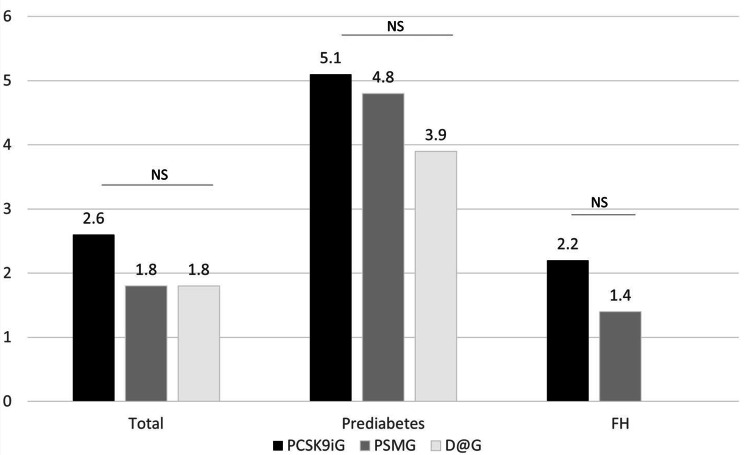
Incidence (%/year) of new onset Diabetes Mellitus according to their original group PCSK9iG: PCSK9i users; PSMG: Propensity score matching group (control group 1); D@G: di@bet.es cohort (control group 2); FH: familial hypercholesterolemia group

Fourteen out of 168 non-DM patients in the PCSK9iG group at baseline developed overt DM (8.3%), representing an incidence rate of 2.6%/y. The incidence in both the PSMG and D@G comparison groups was 1.8%/y (*p* = 0.69 vs. PCSK9iG). Importantly, all 14 patients from the PCSK9iG group that transitioned to overt DM had pre-DM at baseline. The new-onset DM incidence among the 77 pre-DM patients in the PCSK9iG group was 5.1%/y, and this value was 4.8%/y and 3.9%/y among the pre-DM patients in the PSMG and D@G groups, respectively (*p* = 0.922 and 0.682) (Fig. 1).

**Fig. 2:**
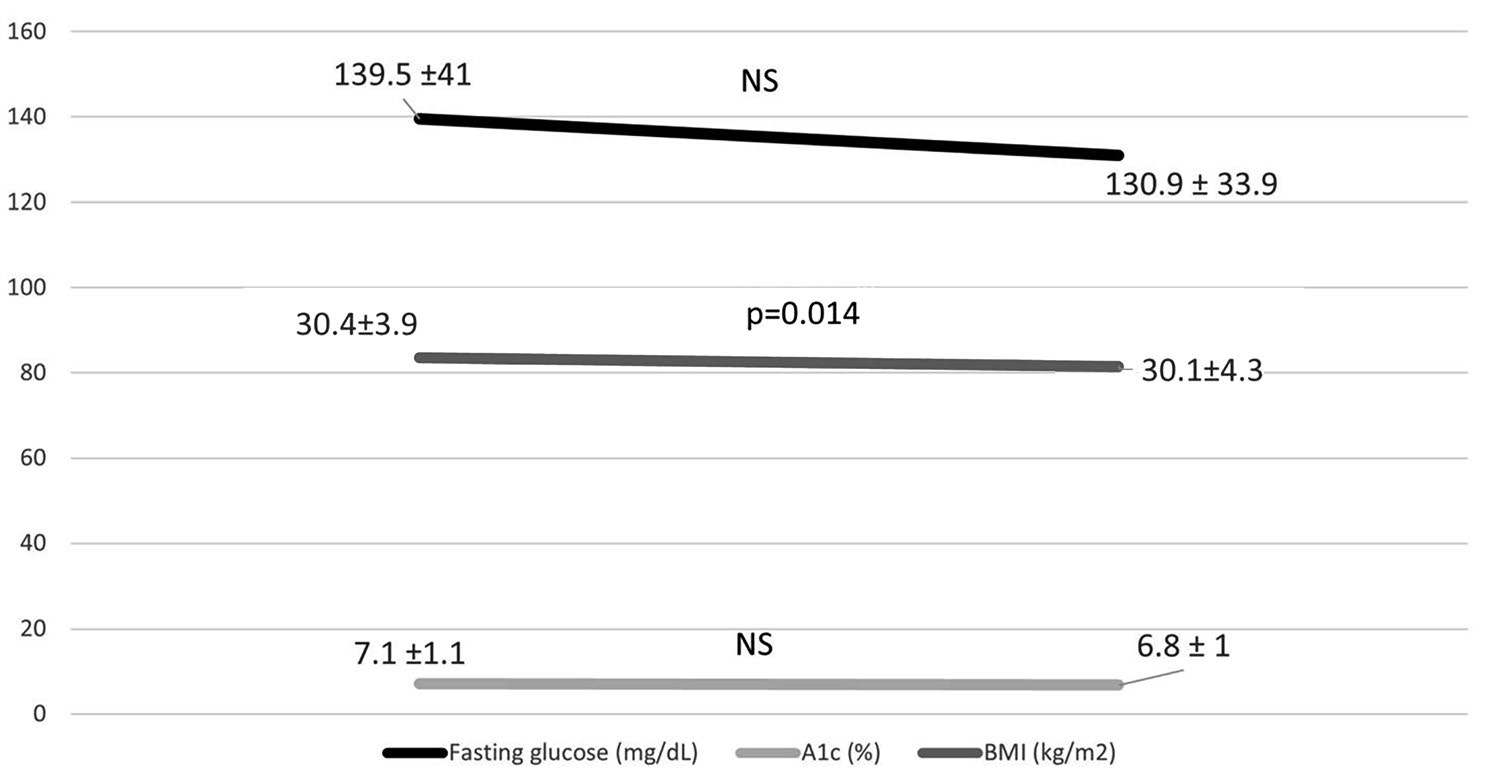
Evolution of fasting glucose, A1c and BMI in DM patients at baseline on PCSK9i Follow-up 3.2 years. Only significant differences were found in BMI A1c: glycated haemoglobin. BMI: body mass index. NS: not significant

Patients with pre-DM who developed DM had higher baseline FG levels than those without diabetes, but there were no differences in the lipid-lowering treatment received, the LDL-C reduction or the time on PCSK9i (Supplementary Table 2). Regarding subjects with DM at baseline, they had a slight but significant decrease of BMI at the end of follow-up, without changes in either FG or A1 (Fig. 2).

As expected from the clinical settings (specialised lipid units), more than 70% of the patients had FH. The incidence of DM was 2.2%/y and 1.4%/y in the PCSK9iG and PSMG FH patients, respectively (*p* = 0.67) (Fig. 1). According to the multivariant logistic regression analysis (Table 3), baseline FG was the only variable significantly associated with the development of DM (OR 1.1; 95% CI: 1.0-1.3; *p* = 0.027).

**Table 3:** Multivariant logistic regression analysis of non-DM PCSK9i-treated patients. Dependent variable: new-onset DM (compared with baseline characteristics)

	OR	CI 95%	sig
Fasting glucose	1.1	1-1.3	**0.027**
BMI	1	0.9–1.2	0.773
Statin	1.5	0.7–35.1	0.791
Ezetimibe	2.4	0.1–40.6	0.531
Age	1	0.9–1.1	0.642
Male sex	1.4	0.2–9.9	0.731
Alirocumab	1	0.03–31.6	0.995
LDL-C reduction at 1y	1	0.9-1	0.590
FH	2.4	0.1–45.1	0.565
Treatment duration (months)	1	1-1.1	0.313

LDL-C: LDL cholesterol; BMI: body mass index; FH: familial hypercholesterolemia

## Discussion

We analysed the effects of treatment with PCSK9i (alirocumab and evolocumab) on the development of *de novo* DM in real-life practice. In general, mild alterations in glycaemic control parameters were recorded during treatment with PCSK9i among the non-DM patients. Approximately a quarter (28.6%) of the patients with normoglycaemia had FG level increases that reclassified them as pre-DM. Although the change in the FG level was statistically significant, it was of little clinical relevance, as it increased from 91 (86-95.5) to 93 (87–101) mg/dL. The incidence of new-onset DM in this cohort was 2.6%/y, which is higher than that of a matched group of patients with metabolic alterations (PSMG, 1.8%/y) and the incidence observed in the general population in the same range of age from the Di@bet.es study (1.8%/y). Although these results did not reach statistical significance (probably because of its low incidence), the difference between the two control groups was consistent. Interestingly, only those patients with baseline pre-DM developed overt DM, suggesting that any diabetogenic effects associated with PCSK9i could play an acceleration effect in DM-prone patients.

Another aspect to be considered is that 70% of the patients in the PCSK9iG had FH. In general, it is believed that FH patients have a lower prevalence of DM [8, 11]; however, the main gene variation [p.(Tyr400_Phe402del)], causing 68% of FH in Gran Canaria Island, is associated with a paradoxical 25% increased prevalence of diabetes [18]. Therefore, the higher incidence of DM in the PCSK9iG group could be expected, as this group of patients had a wide representation of FH when compared to the general population or to metabolic patients, including FH patients from other parts of the country (D@G and PSMG groups, respectively). However, our results do not support this possibility. The new-onset DM in the PCSK9iG and PSMG FH groups was similar to that of the non-FH groups, and there were no differences between them. Moreover, the multilevel multivariate study, which was adjusted for the possible impact of the origin of the subjects on the evolution of their glucose metabolism, showed that the development of DM was only related to baseline FG levels, excluding factors associated with treatment, such as the type of inhibitor, the length of exposure and the percentage reduction in LDL-C levels, as shown in previous studies [12, 13]. As previously mentioned, in the PCSK9iG group, new-onset DM was only diagnosed in subjects who already had baseline pre-DM. These subjects had higher weight gains (although the difference was not significant) than the pre-DM subjects who did not progress to DM, a finding consistent with the results recently published by Merino et al. [10]. This study showed that the diabetogenic effect related to lipid-lowering therapies and LDL-C reduction could be partially mediated by the increase in BMI (38% of the total effect, *p* = 0.003). Interestingly, in the group of patients with DM at baseline in the PCSK9iG group, anthropometric parameters and glucose metabolism control did not worsen, which is probably because of the absence of relevant effects. Moreover, in this group of patients, physicians tend to adapt DM therapies promptly according to clinical practice. Overall, our data suggest that the impact of PCSK9i on glycaemic control, if any, would be moderate, perhaps slightly accelerating the transition to DM in predisposed subjects [19, 20]. Moreover, the efficacy and safety of PCSK9i were demonstrated in clinical trials carried out during their development in patients with and without DM [13, 21]. Neither the ODYSSEY OUTCOMES (alirocumab) nor FOURIER (evolocumab) studies found any deterioration in glycaemic control when compared with placebo, during 2.8 and 2.2 years, respectively [19, 20]. Moreover, most cases of *de novo* DM occurred among subjects with pre-DM, as in our study [20]. The recently published FOURIER-OLE study found no increased risk of *de novo* DM after a median follow-up of five years. The short follow-up time and the fact that most participants in the clinical trials were undergoing statin treatment are factors that could mask a hypothetical risk of DM associated with PCSK9i.

Most meta-analyses published to date have also failed to show an increased risk of DM among patients treated with PCSK9i [22]. In 2020, Chen et al. [14] found an increased risk of DM with alirocumab only when they adjusted for the use of statins, reinforcing the idea that the metabolic repercussions of the inhibitors probably depend to a great extent on the baseline treatment the patient is receiving.

In 2018, Carvalho et al. [15] published a meta-analysis that included more than 68,000 patients with a mean follow-up of 78 weeks. Compared with placebo, subjects treated with PCSK9i experienced a slight but significant increase in FG and A1c levels. However, this did not translate into a significant increase in the incidence of DM, with an association between DM risk and PCSK9i power and duration. These results are consistent with those obtained by Goldman et al. [16] in a recently published real-life study. Hyperglycaemic events were more frequent in PCSK9i users, without higher levels of DM. These effects were observed in the first six months of treatment and were reversible after PCSK9i withdrawal. Analysis according to the type of iPCKS9 indicated that only evolocumab was significantly associated with hyperglycaemia.

Mendelian randomisation studies have analysed gene variants of the *HMGCR, PCSK9 and NPC1L1* genes as a model of the pharmacological action of statins, PCSK9i and ezetimibe. This approach suggested an impact of these three genes on glycaemic metabolism and increased risk of DM, especially among patients who already had altered FG levels [7, 9]. However, it is not known whether the metabolic repercussions of these genetic variants, present from birth, can be assimilated to those of a treatment habitually initiated in adulthood.

The pathophysiological mechanism involved in diabetogenesis associated to PCSK9i is not known. It has been speculated that LDLR upregulation in beta cells could play a role. Higher intracellular cholesterol levels have been related to cell toxicity in animal models [23, 24]. The lower prevalence of DM in FH patients whit less LDLR expression has been postulated to reinforce this theory [8]. Moreover a recent mendelian randomization study including more than 900,000 patients suggest that lower genetically driven LDL-C concentrations are partially mediated by a higher BMI [10].

This study has several limitations; the main ones are its retrospective nature, the small sample size, a relatively short follow-up period and the lack of data availability for some variables of interest, such as A1c, HDL cholesterol or triglycerides levels in non-DM patients. Lifestyle (diet, physical activity), socioeconomic status, race or family background were not taken into account in the PSM and we cannot exclude some impact in DM development. Pre-DM were defined by FG or A1c but glucose tolerance test was not performed, so we could lose some pre-DM patients.

Finally, the initial dose and subsequent adjustments of statins were not assessed although all our patients were on high intensity statins. Moreover, the impact of DM therapy changes was not analysed. The strengths of the study lie on its real life nature, and the comparison with a similar metabolic population and a general population cohort with robust data, which provide a reliable comparison for the main variable of the study: the incidence of new-onset DM.

## Conclusions

Our study has shown that PCSK9i therapy is associated with minute alterations in glucose metabolism control of nonclinical impact. The incidence of new-onset DM was higher in the PCSK9i-treated patients than in both the ad hoc control group and the observed rates in the general population; however, the difference did not reach statistical significance. No changes in glucose parameters were found in subjects with baseline DM. The development of new-onset diabetes was limited to subjects with prediabetes at baseline with higher FG levels and BMI values, so in these cases, closer monitoring of glucose parameters could be important for making an early diagnosis of DM.

In summary, our results do not support a clinically relevant effect of PCSK9i on the risk of DM. In any case, the impact of PCSK9i on glucose homeostasis, if any, should not modify the clinical decision-making process regarding the prescription of these therapies.

### Electronic Supplementary Material

Below is the link to the electronic supplementary material.

Supplementary Material 1

## Data Availability

The PCSK9iG and PSMG datasets used and analysed during the current study are available from the corresponding author upon reasonable request. Dates of the Di@bet.es study are available, but restrictions apply to the availability of these data. They were used under licence for the current study and are thus not publicly available. The data are, however, available from the authors upon reasonable request and with permission of Dr. Josep Ribalta.

## List of Abbreviations

DM: Diabetes mellitus
NODM: New-onset diabetes mellitus
pre-DM: Prediabetes
PCSK9i: PCSK9 inhibitors
LDL-C: LDL cholesterol
FG: Fasting glucose
A1c: Glycated haemoglobin
