# Supplementary material for: Impact of PCSK9 inhibitors in glycaemic control and new-onset diabetes
Source: Cardiovasc Diabetol. 2024 Jan 3;23:4. doi: 10.1186/s12933-023-02077-y (PMC10765818; doi:10.1186/s12933-023-02077-y)
Supplement: Supplementary file 1 — Supplementary Material 1 [file 12933_2023_2077_MOESM1_ESM.docx]

**SUPLEMENTARY TABLES**

**TABLE 1:**

**Baseline characteristics of the PCSK9iG subjects, according to the original hospital.**

|  | **Reus**  **n=124** | **Las Palmas de Gran Canaria**  **n=94** | **p** |
| --- | --- | --- | --- |
| **Age (years)** | 62 (53.5-69) | 62 (54-68) | 0.877 |
| **Sex (male, %)** | 64.5 | 38.3 | **<0.001** |
| **Baseline BMI (kg/m^2^)** | 28.7 (25.6-31.9) | 29.1 (26-31.6) | 0.544 |
| **CVD (%)** | 54.8 | 51.1 | 0.587 |
| **FH (%)** | 66.9 | 75.5 | 0.180 |
| **Ezetimibe (%)** | 62.9 | 62.8 | 1.000 |
| **Statins (%)** | 71.8 | 75.5 | 0.643 |
| **Alirocumab/Evolozumab (%)** | 50/50 | 93.6/6.4 | **<0.001** |
| **Lp(a) (mg/dL)** | 25.8 (9-96.9) | 62 (16-100) | 0.126 |
| **Baseline LDL-C (mg/dL)** | 145.5 (124-180.5) | 172.6 (150,5-201.5) | **<0.001** |
| **LDL-C reduction at 6 months (%)** | 60.9 (44.7-70) | 50.1 (36.3-64.6) | **0.007** |
| **LDL-C reduction at 36 months (%)** | 60.7 (45.2-69.2) | 57.3 (41-71.3) | 0.820 |
| **Fasting glucose (mg/dL)** | 95.5 (87-111) | 107 (97-120) | **<0.001** |
| **Baseline A1c (%) n:151** | 6 (5.6-6.4) | 5.8 (5.5-6.4) | 0.537 |
| **Baseline preDM (%)** | 27.4 | 45.7 | **0.006** |
| **Baseline DM2 (%)** | 16.9 | 30.9 | **0.022** |
| **Final DM2 (%)** | 22.6 | 37.2 | **0.023** |
| **Treatment duration (months)** | 40.5 (14.3-65.2) | 37.5 (25.6-54.6) | 0.970 |

BMI: body mass index; CVD: cardiovascular disease**;** FH: familial hypercholesterolemia; Lp(a): lipoprotein A**;** LDL-C: LDL cholesterol**;** A1c: glycated haemoglobin; preDM: prediabetes**;** DM2: type 2 diabetes mellitus.

**TABLE 2:**

**Characteristics of subjects with pre-DM before starting PCSK9i treatment (n=77) who did/did not develop DM during follow-up.**

|  | **Non-DM n:63 (81.8%)** | **DM n:14 (18.2%)** | **p** |
| --- | --- | --- | --- |
| **Age (years)** | 60.9+/-9.3 | 62.1+/-9.3 | 0.672 |
| **Male sex, (%)** | 42.9 | 57.1 | 0.384 |
| **Baseline BMI (kg/m2)** | 29.5+/-5.2 | 30.7+/-3.6 | 0.441 |
| **Weight gain (kg)** | 1.1 (-1.5-5.3) | 3.1 (-3-4) | 0.876 |
| **CVD (%)** | 46 | 57.1 | 0.559 |
| **FH (%)** | 76.2 | 78.6 | 1.000 |
| **Ezetimibe (%)** | 65.1 | 85.7 | 0.203 |
| **Statins (%)** | 74.6 | 92.9 | 0.173 |
| **Alirocumab /evolocumab (%)** | 74.6/25.4 | 57.1/42.9 | 0.206 |
| **Baseline Lp(a) (mg/dL)** | 27 (11-91) | 17.6 (8.6-76.6) | 0.653 |
| **Baseline LDL-C (mg/dL)** | 178.4+/-51.3 | 176.1+/-42.4 | 0.865 |
| **LDL-C reduction at 6 months (%)** | 59.6 (40.2-67.9) | 54.9 (35.6-74.4) | 0.761 |
| **LDL-C reduction at 36 months (%)** | 55.3 (39.3-69.3) | 57.7 (49.6-63.6) | 0.432 |
| **Fasting glucose (mg/dL)** | 105.5+/-10.1 | 111.8+/-12.7 | **0.047** |
| **Baseline A1c (%)** | 5.8+/-0.3 | 6.2+/-0.3 | **<0.001** |
| **Treatment duration (months)** | 40.4+/-24.5 | 53.3/-28.3 | 0.089 |
| **LDL-C: LDL cholesterol; DM: diabetes mellitus; CVD: cardiovascular disease; HbA1c:** glycated haemoglobin**; FH: familial hypercholesterolemia; BMI: body mass index; Lp(a): lipoprotein A.** | | | |
